# OASA1D-Mediated Tryptophan Enrichment Improves Redox and Ionic Homeostasis Under Salt Stress in Rice

**DOI:** 10.3390/ijms27167236

**Published:** 2026-08-13

**Authors:** Yu Jin Jung, Jin-Young Kim, Hak-Su Kim, Jiyun Go, So Hyun Kim, Jongyeul Baek, Kwon Kyoo Kang

**Affiliations:** 1 Division of Horticultural Biotechnology, Hankyong National University, Anseong 17579, Republic of Korea; yuyu1216@hknu.ac.kr (Y.J.J.); zino@hknu.ac.kr (J.-Y.K.); haksoolove@naver.com (H.-S.K.); shsj1110@hanmail.net (S.H.K.); mkseed@hanmail.net (J.B.); 2 Institute of Genetic Engineering, Hankyong National University, Anseong 17579, Republic of Korea; 3 Department of Bio-Environmental Chemistry, College of Agriculture and Life Sciences, Chungnam National University, Daejeon 34134, Republic of Korea; jy.go2369@gmail.com

**Keywords:** anthranilate synthase, ionic homeostasis, melatonin, *OASA1D*, salt stress, tryptophan

## Abstract

Salinity restricts rice growth by disrupting cellular ion balance and promoting oxidative damage. Although exogenous melatonin can improve rice salt tolerance, whether expansion of the endogenous tryptophan pool enhances melatonin biosynthetic capacity and stress acclimation remains unclear. Here, we investigated a homozygous transgenic rice line constitutively expressing *OASA1D*, a feedback-insensitive D323N variant of the anthranilate synthase α-subunit *OASA1*. The OASA1D-expressing line exhibited strong resistance to 5-methyltryptophan and accumulated approximately twofold more tryptophan than wild-type plants in both shoots and roots under control and 150 mM NaCl conditions. The expanded tryptophan pool was accompanied by a 1.9–2.2-fold increase in endogenous melatonin and elevated expression of the melatonin biosynthetic genes *OsTDC1, OsT5H*, *OsSNAT1*, and *OsASMT1*. Under salt stress, OASA1D seedlings maintained greater shoot and root growth, biomass, and soil–plant analysis development (SPAD) values than wild-type seedlings. OASA1D also showed lower H_2_O_2_ and malondialdehyde accumulation and reduced electrolyte leakage, together with higher superoxide dismutase, catalase, and ascorbate peroxidase activities. Salt-induced expression of *OsDREB2A*, *OsLEA3-1, OsP5CS1, OsWRKY45, OsHKT1;5, OsNHX1*, and *OsSOS1* was enhanced in OASA1D. Consistently, OASA1D shoots accumulated less Na^+^, retained more K^+^, and maintained a higher K^+^/Na^+^ ratio under salinity. Together, these results show that constitutive *OASA1D* expression expands the endogenous tryptophan pool and is associated with enhanced melatonin biosynthetic capacity, antioxidant defence, ionic homeostasis, and salt tolerance in rice.

## 1. Introduction

Rice (*Oryza sativa* L.) productivity is strongly constrained by soil salinity, particularly during germination and early seedling establishment. Salt exposure initially imposes osmotic stress and subsequently causes excessive Na^+^ accumulation, K^+^ depletion, membrane dysfunction, and metabolic disruption. The maintenance of a low cytosolic Na^+^/K^+^ ratio is therefore a major determinant of rice salt tolerance. Identification of the rice salt-tolerance locus *Shoot K^+^ Content 1* (*SKC1*), which encodes the Na^+^ transporter OsHKT1;5, provided direct genetic evidence that restricting Na^+^ transport and maintaining K^+^ homeostasis are central components of salinity tolerance [1]. Salt-induced ionic imbalance is also accompanied by excessive production of reactive oxygen species (ROS), which promotes lipid peroxidation, chlorophyll loss, electrolyte leakage, and growth inhibition. Effective salt acclimation consequently requires coordinated regulation of ion transport, antioxidant defense, and stress-responsive transcription.

Tryptophan is an essential aromatic amino acid and a metabolic precursor of several indolic compounds involved in plant growth and environmental responses. In plants, tryptophan biosynthesis is controlled primarily at the anthranilate synthase reaction, which converts chorismate and glutamine into anthranilate. Anthranilate synthase activity is subject to feedback inhibition by tryptophan, thereby restricting excessive accumulation of the amino acid and its downstream metabolites. Rice contains two anthranilate synthase α-subunit genes, *OASA1* and *OASA2*. Substitution of aspartic acid with asparagine at residue 323 (D323N) in the OASA1 protein produces the feedback-insensitive OASA1D variant. Expression of the corresponding mutant gene, *OASA1D*, reduces the sensitivity of anthranilate synthase to tryptophan, confers resistance to the toxic tryptophan analogue 5-methyltryptophan (5-MT), and markedly increases free tryptophan accumulation [2]. Metabolic profiling of rice expressing *OASA1D* further demonstrated that release of feedback inhibition predominantly expanded the tryptophan pool, with comparatively limited effects on other major metabolites [3,4]. These properties make *OASA1D* a useful genetic system for examining how increased precursor availability influences tryptophan-derived metabolic pathways. Melatonin (*N*-acetyl-5-methoxytryptamine) is synthesized from tryptophan through reactions involving tryptophan decarboxylase (TDC), tryptamine 5-hydroxylase (T5H), serotonin *N*-acetyltransferase (SNAT), *N*-acetylserotonin *O*-methyltransferase (ASMT), and caffeic acid *O*-methyltransferase (COMT). Molecular and biochemical studies in rice have confirmed the functions of SNAT and COMT in melatonin biosynthesis [5,6]. Moreover, overexpression of a rice TDC gene increased endogenous melatonin accumulation, demonstrating that manipulation of an upstream biosynthetic step can alter melatonin production in planta [7]. These observations suggest that both enzyme capacity and precursor availability may determine endogenous melatonin accumulation. Melatonin has been implicated in rice responses to salinity through the regulation of ROS metabolism and ion homeostasis. Exogenous melatonin reduces salt-induced K^+^ efflux by modifying ROS-dependent regulation of plasma-membrane K^+^ transporters and inducing genes involved in K^+^ uptake [8]. Integrated transcriptomic and metabolomic analyses have further shown that melatonin treatment reduces Na^+^ accumulation, limits membrane lipid oxidation, preserves chlorophyll, and activates transcriptional networks involving APETALA2/ethylene-responsive element-binding proteins (AP2/EREBPs), homeobox (HB) proteins, and WRKY transcription factors in salt-stressed rice [9]. At later developmental stages, melatonin also improves antioxidant activity, K^+^ retention, photosynthetic performance, and grain yield under salinity [10]. However, most previous studies have relied on exogenous melatonin application. Furthermore, genetic analyses of melatonin-deficient rice have revealed interactions with other growth-regulatory pathways, including brassinosteroid metabolism, indicating that the contribution of endogenous melatonin to stress tolerance is more complex than that inferred solely from external application [11]. Despite the established feedback-insensitive activity of the OASA1D protein, it remains unknown whether increasing the endogenous tryptophan pool is sufficient to enhance melatonin biosynthetic capacity and whether this metabolic change is associated with improved salinity tolerance. In the present study, we characterized tryptophan and melatonin accumulation in rice constitutively expressing *OASA1D* under control and salt-stress conditions. We further evaluated seedling growth, oxidative damage, antioxidant enzyme activities, stress-responsive gene expression, and Na^+^/K^+^ homeostasis. Our results show that constitutive expression of *OASA1D* expands the endogenous tryptophan pool and is associated with enhanced melatonin biosynthetic capacity and coordinated redox and ion-homeostasis responses during salt stress.

## 2. Results

### 2.1. Functional Verification of OASA1D by 5-Methyltryptophan Resistance and Confirmation of an Expanded Tryptophan Pool Under Salinity

**Figure 1:**
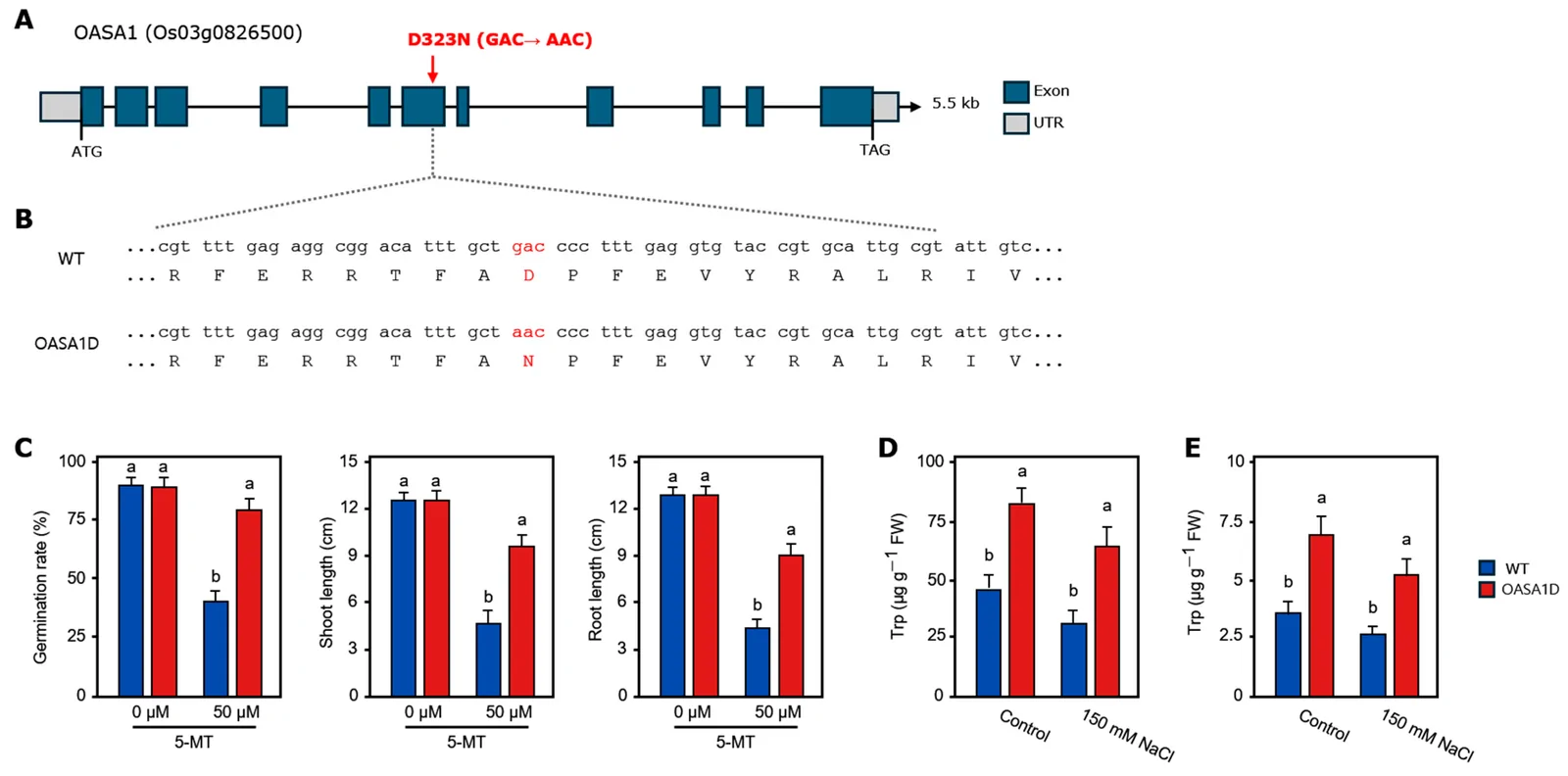
Structural features of *OASA1D*, 5-methyltryptophan resistance, and tryptophan accumulation in *OASA1D*-expressing rice. (**A**) Schematic representation of *OASA1* (Os03g0826500) showing the position of the D323N substitution. Blue and gray boxes indicate exons and untranslated regions, respectively. (**B**) Comparison of the OASA1 and OASA1D nucleotide and deduced amino acid sequences surrounding the D323N substitution. The GAC-to-AAC codon change results in replacement of Asp with Asn at residue 323. (**C**) Germination rate and shoot and primary root lengths of WT and *OASA1D*-expressing seedlings grown in the absence or presence of 50 μM 5-methyltryptophan (5-MT). (**D**,**E**) Tryptophan contents in shoots (**D**) and roots (**E**) of WT and OASA1D plants under control and 150 mM NaCl conditions. Data are presented as means ± SD of three independent biological replicates. For the 5-MT assay, each replicate comprised 30 seeds. Different lowercase letters indicate significant differences among genotype–treatment combinations according to two-way ANOVA followed by Tukey’s multiple-comparison test (*p* < 0.05). FW, fresh weight.

The *OASA1D* transgene encodes the previously characterized D323N substitution in *OASA1* (Os03g0826500), resulting from a GAC-to-AAC codon change (Figure 1A,B). The 5-MT assay was performed as a functional verification of the feedback-insensitive *OASA1D* phenotype and its associated capacity for tryptophan overaccumulation, rather than as an assay of salt-stress resistance. WT and *OASA1D*-expressing seedlings were grown in the absence or presence of 50 μM 5-MT. In the absence of 5-MT, the two genotypes showed comparable germination rates and shoot and root growth. In contrast, 5-MT treatment reduced WT germination to approximately 40%, whereas the *OASA1D*-expressing line retained a germination rate of nearly 80% (Figure 1C). Shoot and primary root elongation were also strongly inhibited in WT seedlings, whereas *OASA1D*-expressing seedlings maintained approximately twofold greater shoot and root lengths under 5-MT treatment. These results confirmed that the line exhibited the expected functional phenotype of feedback-insensitive *OASA1D* expression. The resistant phenotype was accompanied by increased endogenous tryptophan accumulation. Under control conditions, shoot tryptophan content was approximately 1.8-fold higher in OASA1D than in WT, while root tryptophan content was approximately twofold higher (Figure 1D,E). Similar genotype-dependent differences were maintained under 150 mM NaCl treatment, with OASA1D accumulating approximately twice as much tryptophan as WT in both shoots and roots. Although mean tryptophan concentrations declined following NaCl treatment, the effect of salinity was not statistically significant within either genotype. Thus, the 5-MT assay validated the characteristic OASA1D phenotype, whereas the NaCl experiment independently demonstrated that *OASA1D* expression sustained an expanded tryptophan pool in both shoot and root tissues under saline conditions.

### 2.2. OASA1D Attenuates Salinity-Induced Inhibition of Seedling Growth

**Figure 2:**
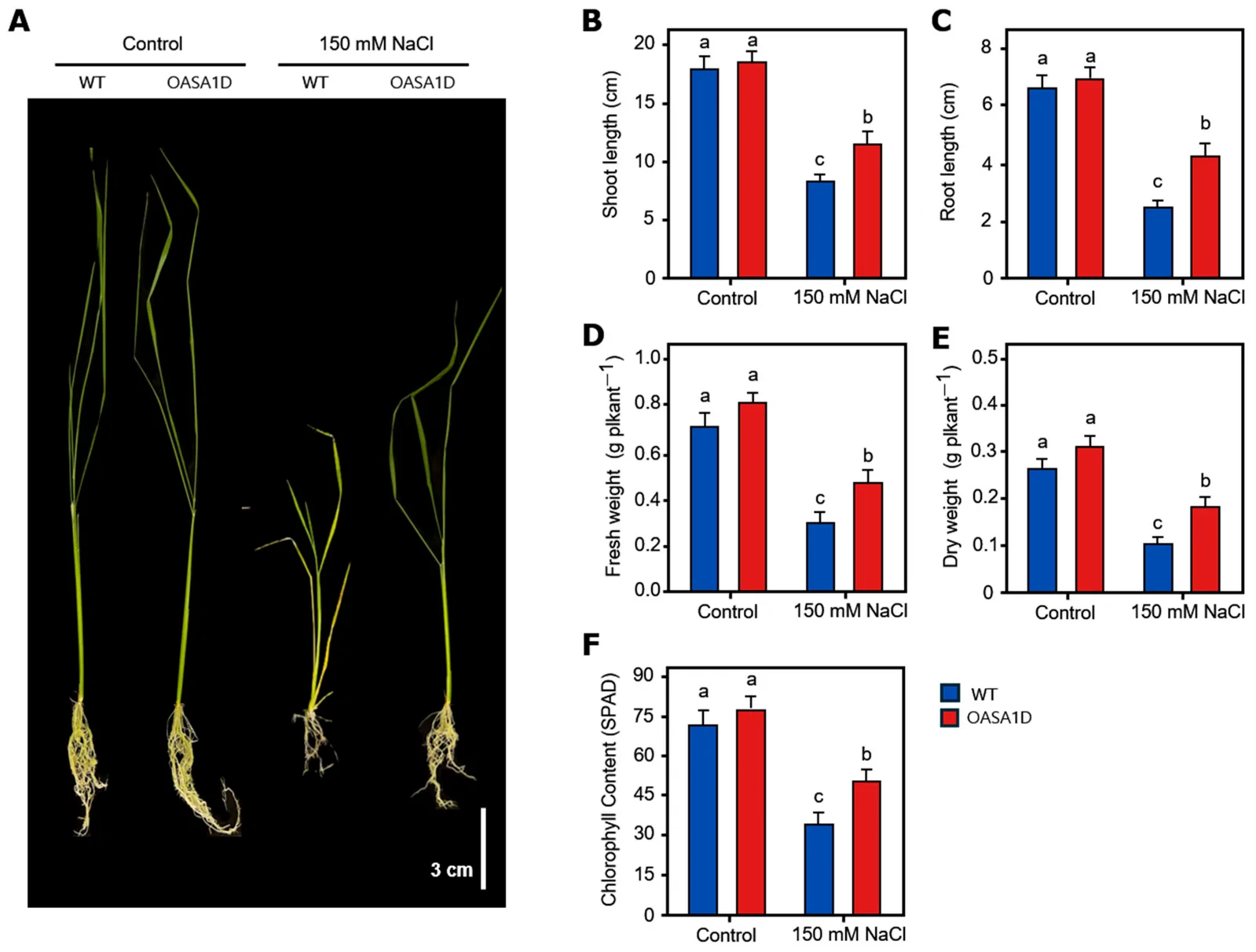
OASA1D alleviates salt-induced growth inhibition and maintains leaf greenness in rice seedlings. (**A**) Representative phenotypes of WT and *OASA1D*-expressing seedlings under control and 150 mM NaCl conditions. Scale bar = 3 cm. (**B**–**F**) Shoot length (**B**), root length (**C**), fresh weight (**D**), dry weight (**E**), and SPAD value (**F**) after 72 h of salt treatment. Fresh and dry weights are expressed on a per-plant basis. Data are presented as means ± SD of three independent biological replicates, with five seedlings per replicate. Different lowercase letters indicate significant differences among genotype–treatment combinations according to two-way ANOVA followed by Tukey’s multiple-comparison test (*p* < 0.05). SPAD, Soil–Plant Analysis Development.

WT and *OASA1D*-expressing seedlings exhibited comparable growth under control conditions, with no significant differences in shoot length, root length, fresh weight, dry weight, or SPAD value (Figure 2A–F). Exposure to 150 mM NaCl markedly inhibited seedling growth in both genotypes; however, the magnitude of inhibition was substantially lower in OASA1D. Under salinity, OASA1D seedlings maintained approximately 38% greater shoot length and 70% greater root length than WT seedlings (Figure 2B,C). Fresh and dry weights were approximately 60% and 80% higher, respectively, in salt-treated OASA1D seedlings than in salt-treated WT seedlings (Figure 2D,E). NaCl treatment also caused more severe leaf chlorosis in WT than in OASA1D seedlings (Figure 2A). SPAD values decreased in both genotypes following salt exposure but remained approximately 43% higher in OASA1D than in WT (Figure 2F). The absence of detectable growth differences under control conditions, together with the consistent advantage of OASA1D under salinity, indicates that *OASA1D* expression primarily attenuated salt-induced growth inhibition and loss of leaf greenness rather than promoting constitutive seedling growth.

### 2.3. Tryptophan Enrichment in OASA1D Is Accompanied by Elevated Melatonin Accumulation and Biosynthetic-Gene Expression

**Figure 3:**
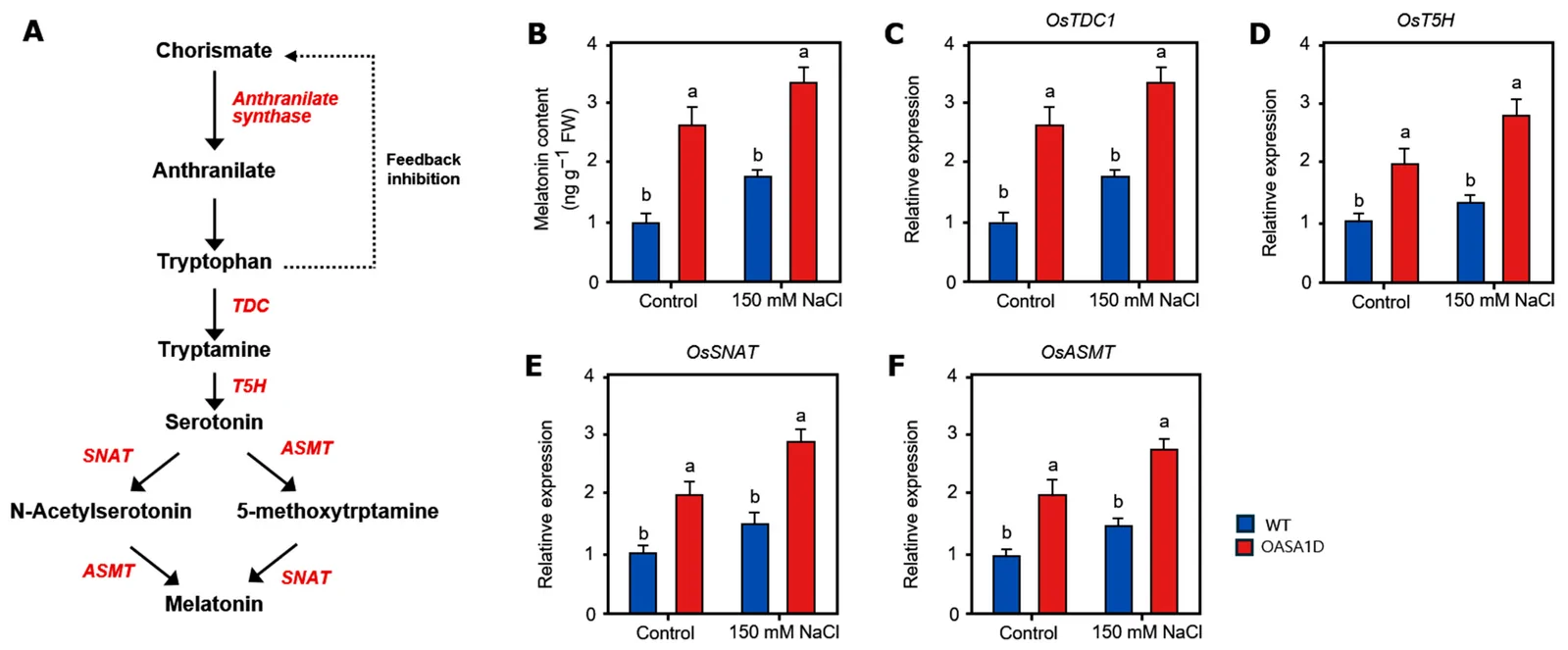
Tryptophan enrichment in OASA1D is associated with elevated melatonin accumulation and melatonin biosynthetic-gene expression. (**A**) Schematic representation of the tryptophan and melatonin biosynthetic pathways. The dotted line indicates feedback inhibition of anthranilate synthase by tryptophan. (**B**) Endogenous melatonin content in shoots of WT and OASA1D plants under control and 150 mM NaCl conditions. (**C**–**F**) Relative transcript levels of *OsTDC1* (**C**), *OsT5H* (**D**), *OsSNAT1* (**E**), and *OsASMT1* (**F**), determined by quantitative reverse-transcription PCR. Transcript levels were normalized to *OsUBQ5* and expressed relative to the WT control. Data are presented as means ± SD of three independent biological replicates. Different lowercase letters indicate significant differences among genotype–treatment combinations according to two-way ANOVA followed by Tukey’s multiple-comparison test (*p* < 0.05). ASMT, N-acetylserotonin O-methyltransferase; FW, fresh weight; SNAT, serotonin N-acetyltransferase; T5H, tryptamine 5-hydroxylase; TDC, tryptophan decarboxylase.

To determine whether the expanded tryptophan pool in OASA1D was associated with increased melatonin accumulation, endogenous melatonin content and the expression of four melatonin biosynthetic genes were analyzed (Figure 3A). OASA1D accumulated approximately 2.2-fold more melatonin than WT under control conditions and approximately 1.9-fold more under 150 mM NaCl treatment (Figure 3B). Within each genotype, the numerical increase in melatonin content following NaCl treatment was not statistically significant. Higher melatonin accumulation in OASA1D was therefore evident before salt exposure and was maintained, rather than specifically induced, under salinity. Expression of *OsTDC1, OsT5H, OsSNAT1*, and *OsASMT1* showed a pattern consistent with melatonin accumulation. Under control conditions, transcript levels of all four genes were approximately twofold higher in OASA1D than in WT (Figure 3C–F). Their expression also remained significantly higher in OASA1D under salt stress, reaching approximately 1.8–2.0 times the levels detected in salt-treated WT plants. Differences between control and NaCl-treated OASA1D plants were not statistically significant. Collectively, these results demonstrate that tryptophan enrichment in OASA1D was associated with higher basal melatonin accumulation and elevated expression of melatonin biosynthetic genes that persisted during salt stress.

### 2.4. OASA1D Limits Oxidative Damage and Preserves Antioxidant Enzyme Activities Under Salt Stress

**Figure 4:**
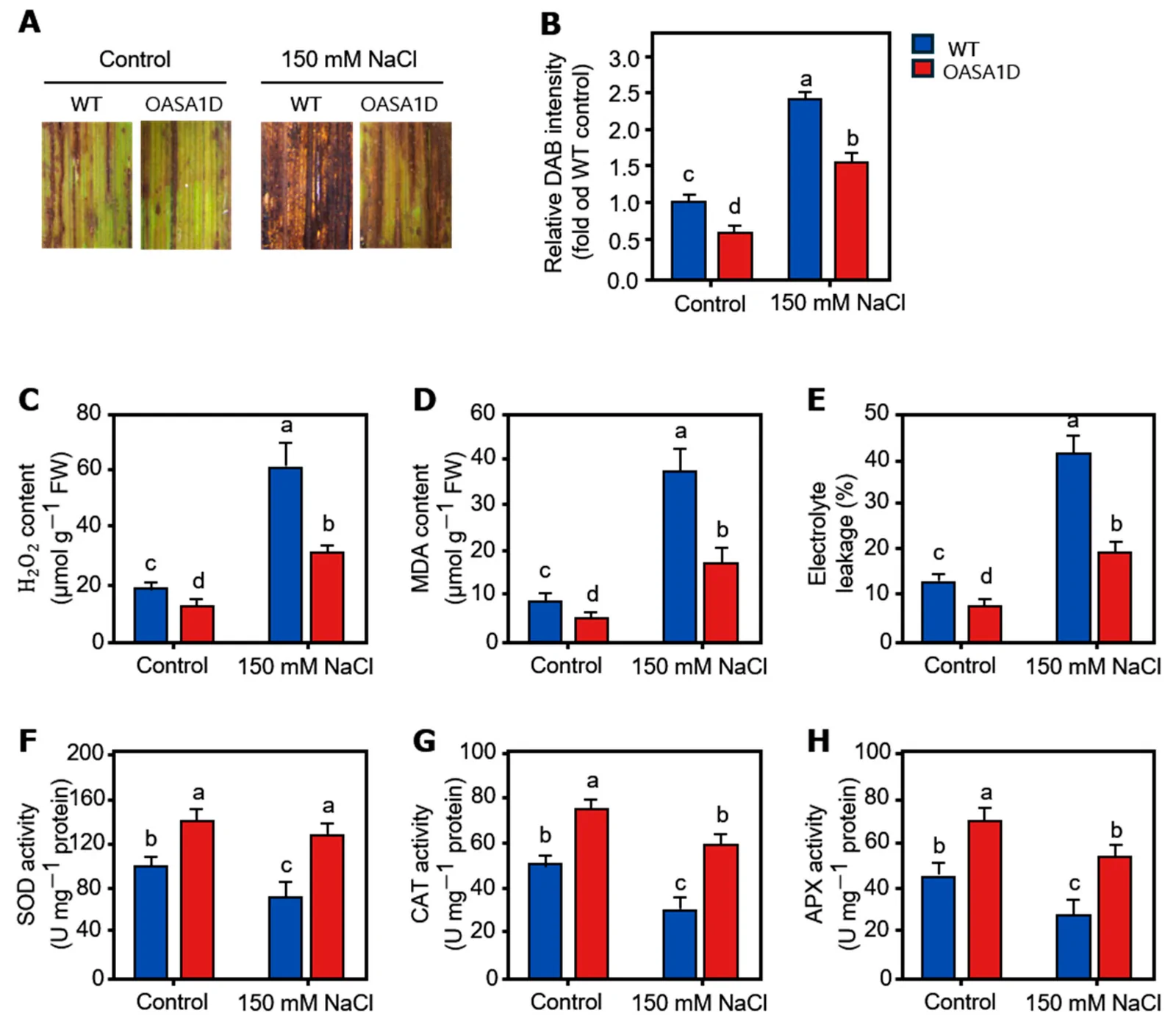
OASA1D enhances antioxidant capacity and limits oxidative and membrane damage under salt stress. (**A**) Representative images of 3,3′-diaminobenzidine (DAB)-stained leaves from WT and OASA1D plants under control and 150 mM NaCl conditions. (**B**) Relative DAB staining intensity normalized to the WT control. (**C**–**E**) Hydrogen peroxide content (**C**), malondialdehyde content (**D**), and electrolyte leakage (**E**). (**F**–**H**) Activities of superoxide dismutase (**F**), catalase (**G**), and ascorbate peroxidase (**H**). Data are presented as means ± SD of three independent biological replicates. Different lowercase letters indicate significant differences among genotype–treatment combinations according to two-way ANOVA followed by Tukey’s multiple-comparison test (*p* < 0.05). APX, ascorbate peroxidase; CAT, catalase; DAB, 3,3′-diaminobenzidine; FW, fresh weight; H_2_O_2_, hydrogen peroxide; MDA, malondialdehyde; SOD, superoxide dismutase.

Salt-induced oxidative injury was evaluated by DAB staining and biochemical determination of H_2_O_2_ and malondialdehyde contents. NaCl treatment markedly increased DAB staining intensity in both genotypes, indicating enhanced H_2_O_2_ accumulation; however, staining was substantially less pronounced in OASA1D leaves than in WT leaves (Figure 4A,B). Quantitative H_2_O_2_ determination supported the histochemical observations. Under salt stress, H_2_O_2_ content was approximately 48% lower in OASA1D than in WT (Figure 4C). Malondialdehyde accumulation, an indicator of membrane lipid peroxidation, was approximately 54% lower in salt-treated OASA1D plants than in salt-treated WT plants (Figure 4D). This reduction in oxidative damage was accompanied by improved membrane stability. Electrolyte leakage increased to approximately 41% in salt-treated WT plants but reached only approximately 19% in OASA1D plants subjected to the same treatment (Figure 4E). OASA1D also exhibited lower H_2_O_2_ content, malondialdehyde accumulation, and electrolyte leakage under control conditions, indicating a lower basal oxidative burden. Activities of superoxide dismutase, catalase, and ascorbate peroxidase were significantly higher in OASA1D than in WT under both control and salt-stress conditions (Figure 4F–H). Although NaCl treatment decreased the activities of all three enzymes, OASA1D retained approximately 1.8-fold higher SOD activity and nearly twofold higher CAT and APX activities than WT under salinity. The maintenance of higher antioxidant enzyme activities in OASA1D was consistent with its reduced ROS accumulation, lipid peroxidation, and membrane injury.

### 2.5. OASA1D Is Associated with Stronger Salt-Responsive Transcription and Improved Ionic Homeostasis

**Figure 5:**
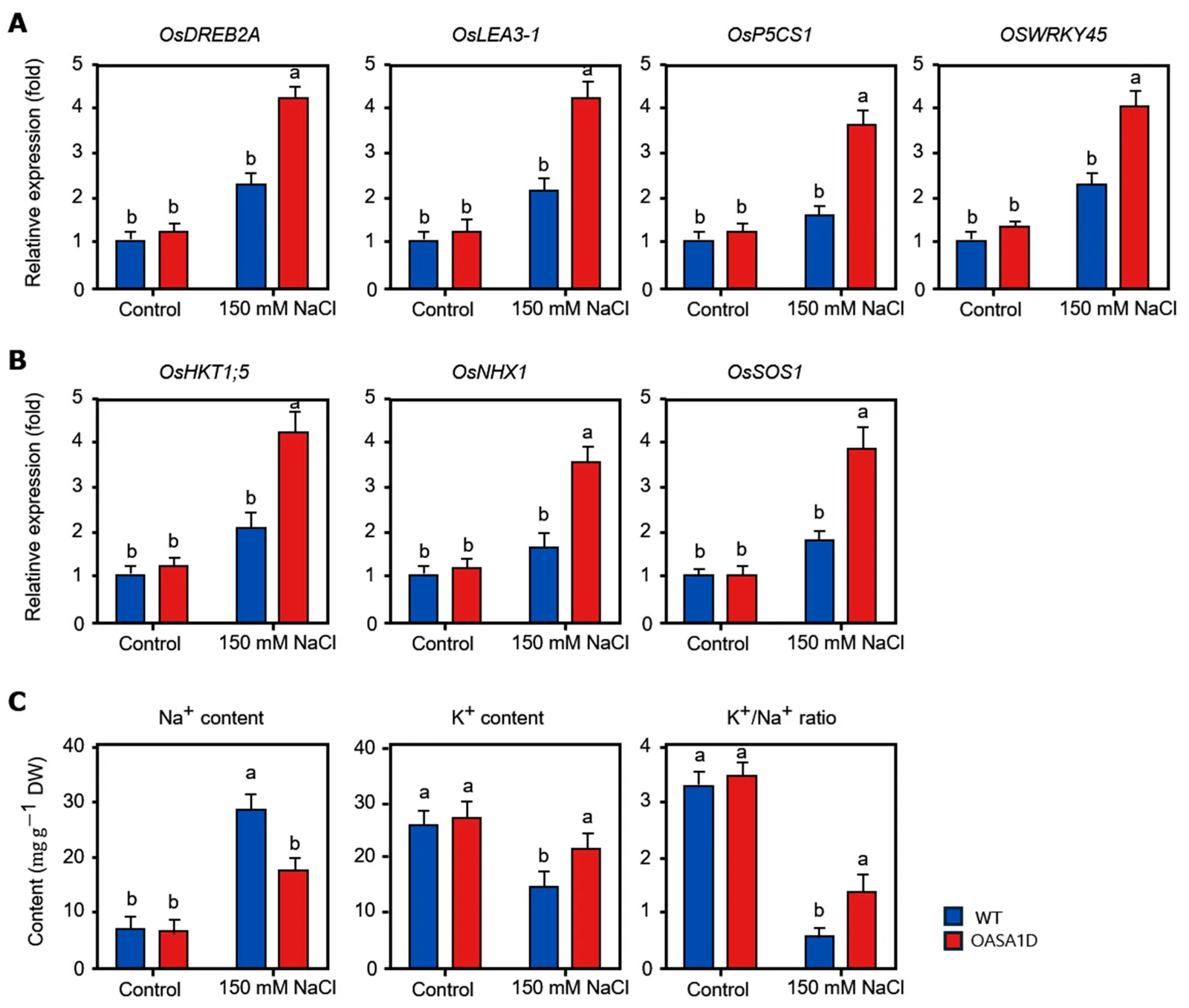
OASA1D enhances salt-responsive gene expression and maintains ionic homeostasis under salinity. (**A**) Relative transcript levels of the stress-responsive genes *OsDREB2A*, *OsLEA3-1*, *OsP5CS1*, and *OsWRKY45* in WT and OASA1D plants under control and 150 mM NaCl conditions. (**B**) Relative transcript levels of the ion-homeostasis genes *OsHKT1;5*, *OsNHX1*, and *OsSOS1*. Transcript abundance was determined by quantitative reverse-transcription PCR, normalized to *OsUBQ5*, and expressed relative to the WT control. (**C**) Shoot Na^+^ content, K^+^ content, and K^+^/Na^+^ ratio in WT and OASA1D plants under control and salt-stress conditions. Data are presented as means ± SD of three independent biological replicates. Different lowercase letters indicate significant differences among genotype–treatment combinations according to two-way ANOVA followed by Tukey’s multiple-comparison test (*p* < 0.05). DW, dry weight.

To determine whether the physiological advantage of OASA1D was accompanied by altered stress-responsive transcription, we analyzed the expression of *OsDREB2A* and *OsWRKY45*, which encode stress-responsive transcription factors; *OsLEA3-1*, which encodes a late embryogenesis abundant protein involved in cellular protection; and *OsP5CS1*, which encodes a key enzyme in proline biosynthesis (Figure 5A). NaCl treatment induced all four genes in both genotypes, but their expression was substantially higher in OASA1D, reaching approximately 1.8–2.2 times the levels detected in salt-treated WT plants. Thus, unlike the elevated basal expression of melatonin biosynthetic genes, enhanced expression of these stress-responsive genes in OASA1D was predominantly associated with salt exposure. A similar genotype-dependent response was observed for the ion-homeostasis genes *OsHKT1;5*, which encodes a Na^+^-selective transporter involved in vascular Na^+^ exclusion; OsNHX1, which encodes a vacuolar Na^+^/H^+^ antiporter; and OsSOS1, which encodes a plasma-membrane Na^+^/H^+^ antiporter. Under control conditions, *OsHKT1;5*, *OsNHX1*, and *OsSOS1* expression did not differ significantly between WT and OASA1D plants. Following NaCl treatment, transcript levels of all three genes were significantly higher in OASA1D than in WT (Figure 5B). In particular, *OsHKT1;5* and *OsNHX1* expression was approximately twofold higher in OASA1D, while *OsSOS1* showed a comparable increase. The transcriptional differences were accompanied by distinct shoot ion profiles. NaCl treatment caused substantial Na^+^ accumulation in WT, whereas OASA1D accumulated approximately 38% less Na^+^ under the same conditions (Figure 5C). Conversely, salt-treated OASA1D plants retained approximately 47% more K^+^ than salt-treated WT plants. Consequently, the K^+^/Na^+^ ratio was more than twofold higher in OASA1D than in WT under salinity. Notably, the K^+^ content and K^+^/Na^+^ ratio of salt-treated OASA1D plants were not significantly different from their respective control values. Together, the stronger induction of ion-homeostasis genes, reduced Na^+^ accumulation, and improved K^+^ retention indicate that *OASA1D* expression was associated with more effective maintenance of ionic balance during salt stress.

## 3. Discussion

The present study demonstrates that expression of the feedback-insensitive *OASA1D* transgene establishes a tryptophan-enriched metabolic state that is associated with improved salt tolerance in rice. OASA1D seedlings exhibited the expected resistance to 5-MT and maintained approximately 1.8–2.0-fold higher tryptophan concentrations than WT in both shoots and roots, irrespective of salt treatment (Figure 1). These findings are consistent with the established role of the D323N substitution in reducing feedback inhibition of OASA1 and increasing carbon flux into the tryptophan biosynthetic pathway [2,3,4]. The magnitude of tryptophan accumulation observed here was smaller than that reported previously in rice callus or mature seeds expressing *OASA1D* [3,4], which may reflect differences in tissue type, developmental stage, growth conditions, or transgene expression. Nevertheless, the maintenance of an expanded tryptophan pool under salinity indicates that the metabolic effect of OASA1D remained functional during stress. The present findings therefore extend previous studies of OASA1D by linking tryptophan enrichment with melatonin accumulation and multiple physiological components of salt tolerance. The enlarged tryptophan pool in OASA1D was accompanied by approximately twofold higher melatonin concentrations and elevated expression of *OsTDC*, *OsT5H*, *OsSNAT*, and *OsASMT* under both control and salt-stress conditions (Figure 3). Tryptophan is the initial precursor of the plant melatonin pathway, and rice TDC, T5H, SNAT, and ASMT catalyze its sequential conversion through tryptamine, serotonin, and N-acetylserotonin [5,6,7]. The simultaneous elevation of the precursor pool, biosynthetic-gene expression, and melatonin content therefore supports the presence of increased melatonin biosynthetic capacity in OASA1D. Genetic enhancement of endogenous melatonin accumulation has previously been associated with increased oxidative-stress tolerance in rice, whereas modification of melatonin catabolism can alter rice responses to salt and other abiotic stresses [12,13]. Notably, NaCl did not significantly increase melatonin content or melatonin biosynthetic-gene expression within either genotype in the present study. OASA1D thus appears to establish a higher basal melatonin state that persists during salt exposure, rather than triggering a salt-specific melatonin burst. However, transcript abundance does not directly represent enzyme activity or metabolic flux. Because isotope-labelled tryptophan tracing was not conducted, the present results do not demonstrate that the additional tryptophan was preferentially directed into melatonin. Tryptophan also supplies serotonin, auxin, and other indolic metabolites; consequently, these compounds may also contribute to the OASA1D phenotype. The physiological protection observed in OASA1D was strongly dependent on the stress condition. WT and OASA1D seedlings were phenotypically comparable under control conditions, whereas OASA1D retained substantially greater root and shoot growth, biomass, and leaf greenness under 150 mM NaCl (Figure 2). This response was accompanied by lower H_2_O_2_ and MDA accumulation, reduced electrolyte leakage, and greater SOD, CAT, and APX activities (Figure 4). Similar antioxidant responses have been observed following exogenous melatonin treatment of salt-stressed rice [8,9,10,14]. Moreover, melatonin-rich transgenic rice exhibits increased resistance to chemically induced oxidative stress [12], and enhanced melatonin biosynthesis in chloroplasts has been associated with decreased ROS accumulation, reduced lipid peroxidation, and improved salt-stress performance in other plant systems [15]. These observations are consistent with the possibility that the higher melatonin status of OASA1D contributes to redox protection. Particularly noteworthy was the presence of higher antioxidant enzyme activities and lower oxidative-damage markers in OASA1D even before salt exposure. This basal difference may represent a metabolically preconditioned redox state that enables OASA1D plants to restrict the early propagation of oxidative damage during salinity. In contrast, the major stress-responsive genes examined here were not constitutively elevated, suggesting that the basal OASA1D phenotype does not simply reflect generalized activation of the entire stress-response program. Because SPAD measurements represent an index of leaf greenness rather than a direct measurement of photosynthetic performance, additional analyses of chlorophyll concentration, gas exchange, and photosystem II efficiency would be required to determine whether OASA1D also preserves photosynthetic function.

In contrast to the constitutive differences in melatonin biosynthesis and antioxidant capacity, the enhanced expression of *OsDREB2A*, *OsLEA3*, *OsP5CS1*, and *OsWRKY45* in OASA1D was predominantly detected after salt treatment (Figure 5A). *OsDREB* proteins regulate subsets of dehydration- and salinity-responsive genes, and overexpression of *OsDREB2A* can enhance dehydration and salt tolerance in rice [16,17]. *OsLEA3-1* is induced by drought, salinity, and ABA and has been associated with cellular protection under water-deficit conditions [18]. *OsP5CS1* encodes a key enzyme in proline biosynthesis and is induced during the osmotic component of rice salt stress [19]. The stronger induction of these genes in OASA1D is therefore consistent with a more responsive transcriptional and osmoprotective system. Nevertheless, proline content, LEA protein abundance, and downstream DREB targets were not measured; their increased transcript levels should consequently be interpreted as evidence of altered stress-responsive transcription rather than direct proof of their individual contributions to tolerance. Particular caution is required when interpreting *OsWRKY45* expression. Dongjin, the japonica cultivar used as the genetic background in this study, carries the *OsWRKY45*-1 allele. Previous allele-specific analyses showed that transgenic lines expressing *OsWRKY45*-1 exhibited no obvious alteration in salt tolerance. By contrast, the indica *OsWRKY45*-2 allele was reported to act as a negative regulator of the salt-stress response [20]. Therefore, the increased *OsWRKY45* expression observed in salt-treated OASA1D plants should not be presented as evidence that *OsWRKY45* positively regulates salt tolerance in this line. Instead, it indicates that the transcriptional response of this stress-associated regulatory network differs between OASA1D and WT. Improved ionic homeostasis represented another major component of the OASA1D salt phenotype. Under salinity, OASA1D accumulated less Na^+^, retained more K^+^, and maintained a K^+^/Na^+^ ratio more than twofold higher than that of WT (Figure 5C). These ion profiles coincided with stronger salt-induced expression of *OsHKT1*;5, *OsNHX1*, and *OsSOS1* (Figure 5B). *OsHKT1*;5 is a Na^+^-selective transporter that protects rice shoots through vascular Na^+^ exclusion, and its transcriptional regulation is an important determinant of K^+^/Na^+^ homeostasis under salinity [1,21,22]. *OsNHX1* contributes to vacuolar Na^+^/H^+^ exchange and intracellular ion compartmentation [23], whereas the conserved rice SOS pathway participates in Na^+^ extrusion and salt adaptation [24]. Coordinated induction of these transport systems could therefore contribute to the lower shoot Na^+^ concentration and improved K^+^ retention in OASA1D. However, the qRT-PCR measurements were obtained from pooled shoot tissue, whereas these transporters function in specific tissues and cellular compartments. Root and xylem-sap ion measurements, tissue-specific expression analysis, and direct Na^+^-flux assays would be required to define the contribution of each transporter.

Collectively, the results support a model in which OASA1D establishes an expanded tryptophan pool that is accompanied by higher basal melatonin biosynthetic capacity and antioxidant activity. When exposed to salinity, OASA1D plants display stronger induction of stress-responsive and ion-homeostasis genes, reduced oxidative and membrane damage, and improved Na^+^/K^+^ balance, ultimately limiting salt-induced growth inhibition (Figure 1, Figure 2, Figure 3, Figure 4 and Figure 5). This model should nevertheless be regarded as associative rather than causal. Only one previously established OASA1D line was examined, and OASA1D may affect multiple branches of tryptophan and indole metabolism [3,4]. Moreover, melatonin metabolism interacts with other hormonal pathways, and melatonin-deficient rice has also shown increased tolerance to some abiotic stresses through altered brassinosteroid homeostasis [11]. Definitive demonstration of a melatonin-dependent mechanism will therefore require independent OASA1D lines, genetic reduction in melatonin biosynthesis in the OASA1D background, exogenous melatonin rescue experiments, and isotope-assisted analysis of tryptophan flux. Finally, the present experiments were conducted during short-term seedling exposure to 150 mM NaCl. Long-term soil-based experiments examining reproductive development and grain yield will be necessary to establish the agronomic relevance of the phenotype. Within these limitations, the results identify OASA1D-mediated tryptophan enrichment as a promising metabolic strategy associated with coordinated redox and ionic protection under salt stress.

## 4. Materials and Methods

### 4.1. Plant Materials and 5-MT Resistance Assay

A previously characterized homozygous transgenic rice line constitutively expressing the feedback-insensitive anthranilate synthase α-subunit gene *OASA1D* and its corresponding wild type (WT; *Oryza sativa* L. ssp. *japonica* cv. Dongjin) were used in this study [2]. The *OASA1D* transgene was derived from *OASA1* (RAP-DB ID: Os03g0826500) by a GAC-to-AAC substitution, resulting in an Asp-to-Asn replacement at amino acid position 323 (D323N). This substitution reduces feedback inhibition of anthranilate synthase by tryptophan. For the 5-methyltryptophan (5-MT) resistance assay, seeds were surface-sterilized with 70% ethanol for 1 min and 2% sodium hypochlorite for 15 min, followed by five rinses with sterile distilled water. Sterilized seeds were germinated on solid half-strength Murashige and Skoog (MS) medium [25] containing either 0 or 50 μM 5-MT. The medium was adjusted to pH 5.8 and solidified with 0.8% agar. Thirty seeds were used per biological replicate, and three independent biological replicates were evaluated. Germination was defined as radicle emergence and was recorded 7 days after sowing. Shoot and primary root lengths were measured 10 days after sowing.

### 4.2. Growth Conditions and Salt Treatment

Seedlings were grown in a controlled-environment chamber under a 14 h light/10 h dark photoperiod, day/night temperatures of 28/22 °C, 60% relative humidity, and a photosynthetic photon flux density of approximately 250 μmol m^−2^ s^−1^. Two-week-old seedlings were transferred to a hydroponic system containing sucrose-free half-strength MS nutrient solution adjusted to pH 5.8 and acclimated for 3 days. The nutrient solution was renewed every 2 days. Salt stress was imposed by adding NaCl to a final concentration of 150 mM, whereas control plants were maintained in the same nutrient solution without NaCl. Shoot samples for metabolite and gene-expression analyses were collected 48 h after treatment. Root samples collected at 48 h were additionally used for tryptophan determination. Growth, oxidative damage, membrane stability, and antioxidant enzyme activities were evaluated after 72 h of treatment. For biochemical and molecular analyses, each biological replicate consisted of tissues pooled from five seedlings, and three independent biological replicates were analyzed. For growth measurements, five individual seedlings were measured per replicate, and their mean was treated as one biological replicate.

### 4.3. Growth and Chlorophyll Measurements

Shoot and primary root lengths were measured after 72 h of salt treatment. Shoots and roots were separated and weighed immediately to determine fresh weight. Dry weight was measured after samples were dried at 70 °C for 48 h. Leaf greenness was evaluated using a SPAD-502 Plus chlorophyll meter (Konica Minolta, Tokyo, Japan). Three readings were taken from the basal, middle, and distal regions of the most recently fully expanded leaf while avoiding the midrib. The mean of the three readings was calculated for each seedling, and the values obtained from five seedlings were averaged to generate one biological replicate.

### 4.4. Tryptophan and Melatonin Quantification

Approximately 100 mg of frozen shoot or root tissue was ground in liquid nitrogen and extracted with 1 mL of methanol containing 0.1% formic acid. Samples were sonicated for 20 min and centrifuged at 15,000× *g* for 15 min at 4 °C. The supernatants were filtered through 0.22 μm polytetrafluoroethylene membranes before analysis. Tryptophan and melatonin were quantified using an Agilent 1290 Infinity HPLC system coupled to an Agilent 6470 triple-quadrupole mass spectrometer (Agilent Technologies, Santa Clara, CA, USA). Metabolites were separated on a ZORBAX Eclipse Plus C18 column (2.1 × 100 mm, 1.8 μm) maintained at 35 °C. Mobile phase A consisted of water containing 0.1% formic acid, and mobile phase B consisted of acetonitrile containing 0.1% formic acid. The flow rate was 0.3 mL min^−1^, and the injection volume was 2 μL. The gradient was programmed as follows: 5% B for 1 min, a linear increase to 95% B over 5 min, maintenance at 95% B for 2 min, and return to 5% B followed by 2 min of column re-equilibration. Detection was performed in positive electrospray ionization mode using multiple-reaction monitoring. The principal transitions were *m*/*z* 205.1 → 188.1 for tryptophan and *m*/*z* 233.2 → 174.1 for melatonin. Quantification was performed using external calibration curves prepared at six concentrations with authentic tryptophan and melatonin standards. Tryptophan was determined separately in shoot and root tissues, whereas melatonin was quantified in shoots. Metabolite concentrations were normalized to tissue fresh weight.

### 4.5. RNA Isolation and Quantitative RT-PCR

Total RNA was isolated from shoots collected 48 h after salt treatment using the RNeasy Plant Mini Kit (Qiagen, Hilden, Germany), including on-column DNase treatment. RNA integrity was evaluated by agarose gel electrophoresis, and RNA concentration and purity were determined using a NanoDrop 2000 spectrophotometer (Thermo Fisher Scientific, Waltham, MA, USA). First-strand cDNA was synthesized from 1 μg of total RNA using the PrimeScript RT Reagent Kit (Takara Bio, Shiga, Japan). Quantitative RT-PCR was performed using a StepOnePlus Real-Time PCR System (Applied Biosystems, Foster City, CA, USA). Each 10-μL reaction contained 5 μL of 2× SYBR Green PCR Master Mix, 0.2 μM of each primer, 1 μL of diluted cDNA, and nuclease-free water. The amplification program consisted of initial denaturation at 95 °C for 2 min, followed by 40 cycles at 95 °C for 15 s and 60 °C for 30 s. Melting-curve analysis was performed to verify the specificity of amplification. No-template and no-reverse-transcription controls were included. Three biological replicates were analyzed, with three technical replicates per biological replicate. The analyzed genes comprised the melatonin biosynthesis genes *OsTDC1, OsT5H, OsSNAT1,* and *OsASMT1*; the stress-responsive genes *OsDREB2A, OsLEA3-1, OsP5CS1*, and *OsWRKY45*; and the ion-homeostasis genes *OsHKT1;5, OsNHX1*, and *OsSOS1. OsUBQ5* was used as the internal reference gene. Relative transcript abundance was calculated using the 2^−ΔΔCt^ method [26], with WT plants under control conditions used as the calibrator. Statistical analyses were performed using ΔCt values. Gene-specific primer sequences and corresponding RAP-DB identifiers are provided in Supplementary Table S1.

### 4.6. Oxidative Damage and Membrane Stability

Hydrogen peroxide accumulation was visualized using 3,3′-diaminobenzidine (DAB) staining [27]. The most recently fully expanded leaves were immersed in 1 mg mL^−1^ DAB solution adjusted to pH 3.8 and incubated for 8 h in darkness. The stained leaves were subsequently heated in 80% ethanol until chlorophyll was completely removed. Leaves were photographed under identical illumination and exposure conditions. Relative DAB staining intensity was quantified using ImageJ software (version 1.54g; National Institutes of Health, Bethesda, MD, USA) and normalized to the WT control. For quantitative H_2_O_2_ determination, approximately 200 mg of fresh leaf tissue was homogenized in 2 mL of ice-cold 0.1% trichloroacetic acid [28]. After centrifugation at 12,000× *g* for 15 min at 4 °C, 0.5 mL of supernatant was mixed with 0.5 mL of 10 mM potassium phosphate buffer (pH 7.0) and 1 mL of 1 M potassium iodide. Absorbance was measured at 390 nm, and H_2_O_2_ content was calculated from an external H_2_O_2_ standard curve and expressed on a fresh-weight basis. Malondialdehyde content was determined using the thiobarbituric acid reaction [29]. Fresh leaf tissue (200 mg) was homogenized in 2 mL of 0.1% trichloroacetic acid and centrifuged at 12,000× *g* for 10 min. An aliquot of 0.5 mL of supernatant was mixed with 1.5 mL of 0.5% thiobarbituric acid prepared in 20% trichloroacetic acid. Samples were heated at 95 °C for 30 min, rapidly cooled on ice, and centrifuged. Absorbance was measured at 532 and 600 nm. MDA content was calculated using an extinction coefficient of 155 mM^−1^ cm^−1^ after correction for nonspecific absorbance at 600 nm. Electrolyte leakage was measured using five uniformly sized leaf discs, 8 mm in diameter, per biological replicate [30]. Leaf discs were rinsed with deionized water and incubated in 10 mL of deionized water for 2 h at room temperature. Initial conductivity (EC_1_) was measured, after which the samples were boiled for 20 min, cooled to room temperature, and measured again to obtain total conductivity (EC_2_). Electrolyte leakage was calculated as follows:

(1) $$\mathrm{Electrolyte}\;\mathrm{leakage}\;\left(\%\right)=\frac{{EC}_{1}}{{EC}_{2}}\times 100$$

### 4.7. Antioxidant Enzyme Activities

Fresh leaf tissue (0.5 g) was homogenized in 5 mL of ice-cold 50 mM potassium phosphate buffer (pH 7.8) containing 1 mM EDTA and 1% polyvinylpyrrolidone. For ascorbate peroxidase extraction, 1 mM ascorbate was additionally included in the extraction buffer. Homogenates were centrifuged at 12,000× *g* for 20 min at 4 °C, and the resulting supernatants were used for enzyme assays. Soluble protein concentration was determined using the Bradford method with bovine serum albumin as the standard [31]. Superoxide dismutase activity was measured based on the inhibition of nitroblue tetrazolium photoreduction [32]. The reaction mixture contained 50 mM potassium phosphate buffer (pH 7.8), 13 mM methionine, 75 μM NBT, 0.1 mM EDTA, 2 μM riboflavin, and enzyme extract. Following illumination for 15 min, absorbance was measured at 560 nm. One unit of SOD activity was defined as the amount of enzyme required to inhibit NBT reduction by 50%. Catalase activity was determined by monitoring H_2_O_2_ decomposition at 240 nm [33]. The reaction mixture contained 50 mM potassium phosphate buffer (pH 7.0), 15 mM H_2_O_2_, and enzyme extract. Activity was calculated using an extinction coefficient of 39.4 mM^−1^ cm^−1^. Ascorbate peroxidase activity was measured from the decrease in absorbance at 290 nm caused by ascorbate oxidation [34]. The reaction mixture contained 50 mM potassium phosphate buffer (pH 7.0), 0.5 mM ascorbate, 0.1 mM EDTA, 0.1 mM H_2_O_2_, and enzyme extract. Activity was calculated using an extinction coefficient of 2.8 mM^−1^ cm^−1^. Enzyme activities were normalized to soluble protein content.

### 4.8. Na^+^ and K^+^ Analysis

Shoot tissues collected after 72 h of salt treatment were dried at 70 °C to a constant weight and finely ground. Approximately 100 mg of dried tissue was digested with 5 mL of concentrated nitric acid using a microwave digestion system. Digested samples were diluted to 25 mL with ultrapure water. Na^+^ and K^+^ concentrations were determined using an Agilent 5110 (Agilent Technologies, Santa Clara, CA, USA) inductively coupled plasma optical emission spectrometer against certified elemental standards. Ion contents were expressed as mg g^−1^ dry weight. The K^+^/Na^+^ ratio was calculated directly from the measured concentrations on a mass basis.

### 4.9. Statistical Analysis

All experiments were conducted using three independent biological replicates. Data are presented as the means ± standard deviation. The effects of genotype, treatment, and their interaction were analyzed using two-way ANOVA, followed by Tukey’s multiple-comparison test. Differences were considered statistically significant at *p* < 0.05. Statistical analyses were performed using GraphPad Prism (version 9.5.1; GraphPad Software, LLC, Boston, MA, USA).

## 5. Conclusions

This study demonstrates that constitutive expression of the feedback-insensitive OASA1D allele establishes a tryptophan-enriched metabolic state in rice and is associated with enhanced tolerance to acute salt stress. OASA1D seedlings maintained higher tryptophan and melatonin contents, together with elevated expression of melatonin biosynthetic genes, under both control and saline conditions. Following treatment with 150 mM NaCl, OASA1D plants retained greater growth and leaf greenness, accumulated less H_2_O_2_ and malondialdehyde, exhibited lower electrolyte leakage, and maintained higher superoxide dismutase, catalase, and ascorbate peroxidase activities than wild-type plants. These responses were accompanied by stronger salt-induced expression of stress-responsive and ion-homeostasis genes, reduced shoot Na^+^ accumulation, improved K^+^ retention, and a higher K^+^/Na^+^ ratio. Collectively, these findings support a model in which release of anthranilate synthase feedback inhibition creates a metabolically preconditioned state that improves redox buffering and ionic homeostasis during salinity stress. However, because the present study was conducted using a single transgenic line and short-term salt treatment at the seedling stage, the direct contribution of melatonin and the agronomic value of OASA1D should be further examined using independent transgenic lines, pathway-specific genetic analyses, metabolic-flux studies, and long-term soil-based salinity experiments.

## Data Availability

The original contributions presented in this study are included in the article/Supplementary Materials. Further inquiries can be directed to the corresponding authors.

## Supplementary Materials

The following supporting information can be downloaded at https://www.mdpi.com/article/10.3390/ijms27167236/s1.
