# Supplementary material for: OASA1D-Mediated Tryptophan Enrichment Improves Redox and Ionic Homeostasis Under Salt Stress in Rice
Source: Int J Mol Sci. 2026 Aug 13;27(16):7236. doi: 10.3390/ijms27167236 (PMC13513127; doi:10.3390/ijms27167236)
Supplement: Supplementary file 1 [file ijms-27-07236-s001.zip › ijms-4482457-supplementary.pdf]

Supplementary Data

Table S1. Gene-specific primers used for quantitative RT-PCR.

Gene	RAP-DB ID	Forward primer (5′–3′)	Reverse primer (5′–3′)	Amplicon (bp)
OsTDC1	Os08g0140300	TTCCAGCCGCTCAACGCCGATGAT	ACATTGGGCAGCACCGGCATGGAC	107
OsT5H	Os12g0268000	ACCCGGACTACAAGCTGCTGCCGT	AGAGCAAGCTGGCGAGCGACACCT	102
OsSNAT1	Os05g0481000	TTTCAGATGCGTCCCCACGGCGTC	TCAAGGACCAAGTTGCCGCAGCCC	122
OsASMT1	Os09g0344500	CGCCGTGGCTTCGCAGTTCAGCTT	TCATTTCCCAACGGGTGCAGCCGT	104
OsDREB2A	Os01g0165000	ACGCGAAGGAAAAGCGATGGCCCT	TTGGACCCCTTGGCTGGCGCTTTC	119
OsLEA3-1	Os05g0542500	AGGCGGCGAAGGACAAGACCTCCA	TGCTTGGCCTGCTCGGTCTTCTCG	112
OsP5CS1	Os05g0455500	TTGGCTTTGGGCAGGGTTGGAGCT	TCGCCCCACTCCAACAGCACCTGA	105
OsWRKY45	Os05g0322900	GATGTCACGTCCTCCCTGCACT	CGGTGATCAAAAGCTCAAACCC	117
OsHKT1;5	Os01g0307500	TGCATCACGGAGCGGCGGCAAATC	ACCCCGTGCTGAACCCCACATTGC	109
OsNHX1	Os07g0666900	TGCTCCTCACCAAGCCGACCCACA	GCCCGCCAAACATCGGTCGCATCA	84
OsSOS1	Os12g0641100	TGTTGCTCTGGGCCTTGCTTTTGGA	AGCTGACAGCAAGAGTAAGCGCGAT	101
OsUBQ5	Os01g0328400	GGCCAACCACTTCGACCGCCACTA	TTCCGCCCCCAAAGAACAGGAGCC	99
